# Supplementary material for: Patterns of Intron Gain and Loss in Fungi
Source: PLoS Biol. 2004 Nov 30;2(12):e422. doi: 10.1371/journal.pbio.0020422 (PMC532390; doi:10.1371/journal.pbio.0020422)
Supplement: Table S1 — Also available at http://genes.mit.edu/NielsenEtAl/. (4.3 MB ZIP). [file pbio.0020422.st001.zip › NielsenEtAl/html/833.html]

AN1050.1.NCU09646.1.MG10700.1.FG04243.1


```
 CLUSTAL W (1.82) Multiple Sequence Alignments - Introns Inserted


Sequence 1: MG10700.1	416 aa
Sequence 2: FG04243.1	416 aa
Sequence 3: NCU09646.1	415 aa
Sequence 4: AN1050.1	417 aa
Alignment Length: 419 aa
Number Identitical Residues: 254 aa
Alignment Score (without introns) 11537


MG10700.1 	MAVERLGSILKHLTPGN-ALSTI2TSKNDDDIVITYAARTPLGKARKGGFKDTSLEYMVF
NCU09646.1	MAVERLGSILKHLTPGT-ALSNI~TAKNPDDIVITLAIRTPLTKAKKGGFKDTSLEYLAY
FG04243.1 	MAAERIGSIIKHLAPGS-ALNSI2QSKNPDDIVITLAVRTPLTKAKKGGFKDTSLEYMVY
AN1050.1  	MATERLNSILSHLRPGNKGVAAI2TQKNPDDVVITLSLRTPLTKARKGGFKDTELDYLVY
          	**.**:.**:.** **...:  *   ** **:*** : **** **:*******.*:*:.:

MG10700.1 	ALLDQVRQRSKIDPSLVEDICMGN~VSDGKAAYKLRAAALAAGFPNTHSASSVNRFCSSG
NCU09646.1	ATLKEVKERCNMDPALVEDIAMGN0VSDGKAAYKLRAAALAAGFPNTAGAYSLNRFCSSG
FG04243.1 	AILKQVRERSNLDPALVEDICLGN0VSDVKAAYKVRASALAAGFPNTAGASSINRFCSSG
AN1050.1  	ALLKEVIAKSQIDPNLIEDVCLGN~VNDGKAAYLVRAASLAAGIPHTAGASSVNRFCSSG
          	* *.:*  :.::** *:**:.:** *.* **** :**::****:*:* .* *:*******

MG10700.1 	LKATADIAHAISNGSIEVGIALGAESMSIGGDALDQPFDEDVTSQNQESKDCMMPMGWTS
NCU09646.1	LKAVADIAHAISCGSIEVGIAMGAESMSVGGDALEQPFDEAVTSQSQESADCMQPMGWTS
FG04243.1 	LKATADIAHSISNGSIEVGIAMGAEQMSIGGDALDKPFDEAVTSQSQEAVDCMQPMGWTS
AN1050.1  	LKAVQDIANQISLGAIDIGVAVGAEIMSAGGDRLNKPFNEEVLK-NQEAADCMQPMGQTS
          	***. ***: ** *:*::*:*:*** ** *** *::**:* * . .**: *** *** **

MG10700.1 	ENVSSDFGISREEMDKFAALSFQKAEKAQNAGWFDDEIVPIRTKVKGP-DGEVKEVTLTK
NCU09646.1	ENVSRDFGITREEMDKYAAESFQRAERAQKAGLFDDEIVPIKTRVKDA-NGEWKDVTLTK
FG04243.1 	ENVSKDFGVTREMMDKFAAGSFQKAEAAQKAGWFADEIVPITTKVKGP-DGQEKEVTLTQ
AN1050.1  	ENVGKDFNISRAQQDVYAAESFRRAEVAQKAGWFDDEIVPITTRVKDPKTGEERSVTLTK
          	***. **.::*   * :** **::** **:** * ****** *:**... *: :.****:

MG10700.1 	DEGIRPGTTAESLGKIRAAFPQWGSTTTGGNASQVTDGAAAVLLMKRSTANKLGQPIMAK
NCU09646.1	DEGIRPGTTAESLSKIRVAFPQWGPTTTGGNASQVTDGAAAILLMKRSTAVKLGQPILGK
FG04243.1 	DEGIRPGTTAESLSKIRAAFPQWGGATTGGNASQITDGAAAVLLMKRSTAIKLGQPIMAK
AN1050.1  	DDGVRYGTTVESLSKIRPAFPQFGDKSTGGNSSQVTDGAAAVLLMRRSKAIELGQPILAK
          	*:*:* ***.***.*** ****:*  :****:**:******:***:**.* :*****:.*

MG10700.1 	YVGSTTAGLAPRIMGIGPTVAIPKLLAKHGITLADCDVVEINEAFASMAVYCRDNLGLDA
NCU09646.1	YVGSTVAGLAPRIMGIGPSIAIPKLLNQYGLTLNDVDVVEINEAFASMAVYCRDKLQLDW
FG04243.1 	YVGSTVAGLAPRIMGIGPSVAIPKLLAQHNISMDDIDIVEINEAFASMAVYCQDKLGLTS
AN1050.1  	FVGATVAGVPPRVMGIGPTAAIPKLLSKFNLDKNDVDIYEINEAFASMAVYCVEKLGLDH
          	:**:*.**:.**:*****: ****** :..:   * *: ************* ::* *  

MG10700.1 	ETKMNPRGGAIALGHPLGATGTRQIVTGLSECRRTGKKILLTSMCIGTGMGMAGLFVNEQ
NCU09646.1	N-KMNPRGGAIALGHPLGATGARQIVTGLAECRKSGKKVLLTSMCIGTGMGMAGLFVNEQ
FG04243.1 	E-KLNPRGGAIALGHPLGATGARQIVTGLAECRRQKKKMLLTSMCIGTGQGMAGLFVNEQ
AN1050.1  	A-KVNPRGGAIALGHPLGATGARQIATILSEARRTKAKVLVTSMCIGTGQGMAGLFVNEQ
          	  *:*****************:***.* *:*.*:   *:*:******** **********

MG10700.1 	-
NCU09646.1	-
FG04243.1 	V
AN1050.1  	V
          	
```
